# Identification of potential novel proteomic markers of *Leishmania* spp.-derived exosomes

**DOI:** 10.3389/fcimb.2024.1354636

**Published:** 2024-02-19

**Authors:** Alonso da Silva Lira Filho, Andrea Lafleur, Marcelo Marcet-Palacios, Martin Olivier

**Affiliations:** ^1^ Department of Microbiology and Immunology, McGill University, Montréal, QC, Canada; ^2^ Infectious Diseases and Immunity in Global Health Program, Research Institute of the McGill University Health Centre, Montréal, QC, Canada; ^3^ Department of Medicine, Alberta Respiratory Centre, University of Alberta, Edmonton, AB, Canada; ^4^ Department of Biological Sciences Technology, Laboratory Research and Biotechnology, Northern Alberta Institute of Technology, Edmonton, AB, Canada

**Keywords:** *Leishmania*, cutaneous leishmaniasis, proteomics, extracellular vesicles, exosomes

## Abstract

**Introduction:**

Extracellular vesicles (EVs) are heterogenous cell-derived membrane-bound structures which can be subdivided into three distinct classes according to distinct morphological characteristics, cellular origins, and functions. Small EVs, or exosomes, can be produced by the protozoan parasite *Leishmania* through the evolutionarily conserved ESCRT pathway, and act as effectors of virulence and drivers of pathogenesis within mammalian hosts. Techniques for the identification of EVs of non-mammalian origin, however, remain inaccurate in comparison to their well-characterized mammalian counterparts. Thus, we still lack reliable and specific markers for *Leishmania*-derived exosomes, which poses a significant challenge to the field.

**Methods:**

Herein, we utilized serial differential ultracentrifugation to separate *Leishmania*-derived EV populations into three distinct fractions. Nanoparticle tracking analysis and transmission electron microscopy were used to validate their morphological characteristics, and bioinformatic analysis of LC-MS/MS proteomics corroborated cellular origins and function.

**Discussion:**

Proteomic data indicated potential novel proteic markers of *Leishmania*-derived exosomes, including proteins involved in endosomal machinery and the ESCRT pathway, as well as the parasitic phosphatase PRL-1. Further investigation is required to determine the specificity and sensitivity of these markers.

## Introduction

The trypanosomatid *Leishmania* is a protozoan parasite that is transmitted to various mammalian hosts through the bloodmeal of the female phlebotomine sandfly (World Health Organization, 2023). *Leishmania* infection can lead to various diseases collectively called leishmaniases, for which clinical presentation varies immensely depending on the causative species and host factors (World Health Organization, 2023). While Cutaneous leishmaniasis usually presents as self-healing cutaneous lesions, parasitic metastasis to the nasopharyngeal mucosal regions causes severe and disfiguring mucocutaneous leishmaniasis. The most severe form, visceral leishmaniasis, is characterized by systemic infection and is lethal in nearly all untreated cases (World Health Organization, 2023). Despite over half the world’s population being at risk of contracting the infection, primarily in low-income countries, leishmaniasis has been classified as a neglected tropical disease (NTD) by the World Health Organization due to a significant lack of investment in the research and development of therapeutics (World Health Organization, 2023).

The term extracellular vesicles (EVs) encompasses exosomes, ectosomes, and apoptotic bodies – each of which has a distinct cellular origin, diameter, function, and physical properties. Exosomes – the smallest EVs (30-150 nm) – are of endosomal origin and are ubiquitously produced by eukaryotic and prokaryotic cells (Johnstone et al., 1987). They have been shown to play a role in many physiological and pathological functions, including cell-cell communication, immunological responses, renal pathologies, cancer, and infectious diseases, among others (Johnstone et al., 1987; Turpin et al., 2016; Fernandez-Prada et al., 2018; Skog et al., 2008; Hosseini et al., 2013). The latter is particularly of interest in the context of leishmaniasis, for which exosomes produced by parasites themselves are involved in immunopathogenesis and are a major determinant of disease outcome (Georgiadou et al., 2015).

Exosomes are produced by *Leishmania* spp. in the midgut of the phlebotomine sandfly vector, while parasites are concentrated and packaged into the insect’s infectious inoculum (Bates, 2007; Georgiadou et al., 2015). During the female sandfly’s bloodmeal, leishmanial exosomes are co-egested alongside the parasite into the mammalian host’s skin, immediately exerting effects on surrounding tissues by inhibiting innate immune processes and ultimately exacerbating cutaneous pathology (Atayde et al., 2015). This process is partially driven by the surface metalloprotease GP63 – a key leishmanial virulence factor which permits motility through the extracellular matrix and abrogates leishmanicidal macrophage function by way of inhibiting oxidative burst, among other roles (Olivier et al., 2012; Hassani et al., 2014). Thus, the study of leishmanial exosomes and their role in parasitic survival and virulence is a growing field of study and is essential to understanding the progression of disease.

However, the isolation and identification of leishmanial exosomes continue to pose a challenge in the field, as is the case with most EVs of non-mammalian origin. Although markers for mammalian exosomes, such as CD63, CD9, and CD81, have been identified in recent studies, the lack of homologous proteins in *Leishmania* spp. presents a significant challenge for the precise identification of leishmanial exosomes (Kowal et al., 2016). Further, while Heat Shock Proteins, particularly HSP83 and HSP70, are used as predictive markers of *Leishmania* spp.*-*derived exosomes, they display a lack of specificity to this vesicle class and have been identified across all EV populations, introducing potential error due to misclassification and improper utilization of extracellular vesicles of non-exosomal origin (Santarém et al., 2013). Herein, we aimed to address this knowledge gap by identifying potential novel biomarkers of leishmanial exosomes through proteomic and bioinformatic studies of all subclasses of EVs, obtaining subpopulations by adapting Kowal et al.’s methodology for serial differential ultracentrifugation of mammalian EVs to protozoa.

## Materials and methods

### Parasites cell culture

*Leishmania major* Friedlin strain (MHOM/IL/81/FN) promastigotes were cultured in SDM (Schneider’s Drosophila Medium) supplemented with 10% heat-inactivated fetal bovine serum (FBS) and 5mg/mL HEMIN at 25°C. Parasites were passaged to fresh culture media biweekly (every 3-4 days) to maintain logarithmic growth.

### Extraction and fractionation of *L. major*-derived vesicles

Methods used to extract *L. major* extracellular vesicles and perform their fractionation were adapted from Kowal et al. and as previously described by our lab (Kowal et al., 2016; Hassani et al., 2011; Vucetic et al., 2020). Modifications to methodology published Kowal et al. were made to account for protozoan rather than mammalian cell culture and relevant centrifugation speeds for *Leishmania* (Kowal et al., 2016; Hassani et al., 2011; Vucetic et al., 2020). Briefly, 800 mL of late log phase (Day 7-9) parasite culture was centrifuged and pelleted at 300 x g (RCF). Parasites were resuspended and washed three times with PBS before being centrifuged and pelleted at 300 x g (RCF) after each washing. The resulting pellet was resuspended in FBS-free RPMI 1640 medium without phenol-red (Life Technologies) at a concentration of 1–4 × 10^8^ parasites/mL, then incubated at 37°C with low agitation (40-60 RPM) in a shaking incubator for 4 hours. Following incubation, the culture was centrifuged at 300 x g (RCF) for 5 minutes to pellet parasites and separate the supernatant from cells. Next, the vesicle-enriched supernatant was collected and centrifuged at 2,000 x g (RCF) for 10 minutes. The resulting pellet (2K) was collected and resuspended in exosome buffer (137 mM NaCl, 20 mM Hepes pH 7.5), while the supernatant was collected for subsequent centrifugation. Finally, the supernatant was transferred to open-top thin wall polypropylene tubes (16 x 102 mm) and centrifuged at 10,000 x g (RCF) for 30 minutes at 4˚C using a Beckman Coulter Optima XPN-90™ ultracentrifuge and a swing rotor (SW32.Ti). The resulting pellet (10K) was collected and resuspended in an exosome buffer. The remaining supernatant was centrifuged at 100,000 x g (RCF) for 1 hour at 4°C, and the resulting pellet (100K) was resuspended in approximately 250 µL of exosome buffer. All samples were stored at -80°C until further analysis. Protein concentrations of the fractionated samples were determined with a Micro BCA Protein Assay Kit according to the manufacturer’s protocol (Thermo Scientific; catalog number: 23235). Extraction and fractionation of EV populations was performed in biological triplicates, with each replicate originating from a distinct culture of *L. major* from experimental procedures performed on different days. All downstream assays were performed on each of the three biological replicates.

### Nanoparticle tracking analysis

*L. major*-derived vesicle preparations (2K, 10K, and 100K fractions) were analyzed by NTA using an LM-10 NanoSight machine in the laboratory of Dr. Janus Rak at the Research Institute of the McGill University Health Centre. For the determination of particle size and concentration, three sequential 30-second videos were acquired for each of the 3 biological replicates per fraction, using the default settings of the instrument. Exosome buffer was used as the negative control.

### Transmission electron microscopy

For negative staining, vesicle preparations (2K, 10K, and 100K fractions) from each biological replicate were coated directly on Formvar/Carbon grids, fixed with 1% glutaraldehyde in 0.1 M sodium cacodylate buffer for 1 min, and stained with 1% uranyl acetate for 1 min. Formvar grids covered with isolated vesicles were visualized in the FEI Tecnai 12 120 kV transmission electron microscope. Images were taken with the AMT XR-80C CCD Camera System (Facility for Electron Microscopy Research, McGill University).

### LC-MS/MS proteomic analysis

Proteins from the 2K, 10K, and 100K fractions of *L. major*-derived vesicles from each biological replicate were precipitated with 15% trichloroacetic acid (TCA) and acetone before being digested with trypsin at a concentration of 2 ng/ml. Protein digests were then resolubilized under agitation for 15 minutes in 10 mL of 0.2% formic acid, and desalting/cleanup was performed using C18 ZipTip pipette tips (Millipore, Billerica, MA). Eluates were dried in a vacuum centrifuge and resolubilized under agitation for 15 min in 10 mL of 2% ACN/1% formic acid. Liquid chromatography-tandem mass spectrometry (LC-MS/MS) was performed at the Institut de Recherches Cliniques de Montréal (IRCM, University of Montreal). The column was installed on the Easy-LLC II system (Proxeon Biosystems, Odense, Denmark) and coupled to the LTQ Orbitrap Velos (ThermoFisher Scientific, Bremen, Germany) equipped with a Proxeon nanoelectrospray ion source.

### Bioinformatic analysis

LC-MS/MS data sets were searched with Mascot 2.2 (Matrix Science) against the NCBI *Leishmania major* database (ID: 5664) and UNIPROT *Leishmania major* database (ID: LEIMA) (Apweiler et al., 2004). The mass tolerances for precursor and fragment ions were set to 10 ppm and 0.6 Da, respectively. Trypsin was used and was set to an allowance of up to 2 missed cleavages. Biological triplicates of separately analyzed sets of MS/MS data were used to calculate the peptide count values using Scaffold Software, and normalized in-software using TIC area values (Searle, 2010). Scaffold Software analyzed mascot output files, and hits for further analyses were considered as a minimum of 2 peptides, a peptide threshold of 80%, and a protein identity of 95%. Quantitative analysis of protein expression was performed with Scaffold software using ANOVA for statistical analysis. Differential expression was defined by protein expression levels that were at least 2 standard deviations from the mean expression value derived from all LC-MS/MS samples and a p value inferior to 0.05 (Searle, 2010). Gene Ontology (GO) annotations of identified proteins were obtained using PANTHER database (https://www.panther-db.org) (Mi et al., 2019). Protein-protein interaction (PPI) networks of the identified proteins were generated using String database (https://www.string-db.org) with the highest confidence of interaction (>0.900), showing only connected proteins and using an MCL inflation parameter of 3 (Szklarczyk et al., 2023). Heat maps were generated using GraphPad Prism, plotting Z score expression values as per $Z=\frac{\left(MeanPeptideCount_{Group}\right)-\left(MeanPeptideCount_{Experiment}\right)}{StDev_{Experiment}}$ .

### All-atom scale exosome representation

Software and methods used to reconstruct the all-atom scale exosome were carried out as previously described with some modifications (Sun et al., 2019). Briefly, a lipid bilayer sample was used to coat an isosphere built using Blender version 3.3.0 (https://www.blender.org/). The size of the isosphere was such as to accurately generate a lipid bilayer with an outer diameter of 100 nm (**Figure 6**). Human exosome markers were selected using previous references (Shaabani et al., 2023). Protein databank accessions were: 1KPP (TSG101), 1ZOA (GTPase), 2OER (ALIX), 2QGO (hsp90) and 3HS3 (hsp70). All leishmanial structures shown were predicted using the Alphafold software (https://alphafold.ebi.ac.uk/), as atomic structures for leishmanial exosome markers have not yet been determined 11/22/2023 1:54:00 AMa (Varadi et al., 2022). Full all-atom scale render and sphere size validation were performed using PyMol version 2.5.4 (https://pymol.org/). Additional layers were rendered in Photoshop version 24.7.0.

### Statistical analysis

Statistical analyses of proteomic data were performed using uncorrected ANOVA for multiple comparisons, and p-values less than 0.05 were considered statistically significant. The data sets were analyzed using GraphPad Prism^®^ software, MS Excel^®^, and Scaffold^®^ (Version 5.1.0).

## Results

### Serial differential ultracentrifugation separates *Leishmania* spp.-derived extracellular vesicles into three morphologically diverse populations

To identify potential new markers for *Leishmania* spp.*-*derived exosomes, we adapted the methods used by Kowal et al., 2016, whereby preparations of extracellular vesicles (EV) are fractionated into three distinct groups using serial differential ultracentrifugation at 2,000 x g (2K), 10,000 x g (10K) and 100,000 x g (100K) – corresponding to the relative centrifugal forces required to pellet apoptotic bodies, ectosomes and exosomes, respectively. This approach enabled us to isolate the three major classes of EVs produced by the parasite, facilitating the characterization and differentiation of the exosome-enriched fraction (100K) from the other fractions.

**Figure 1:**
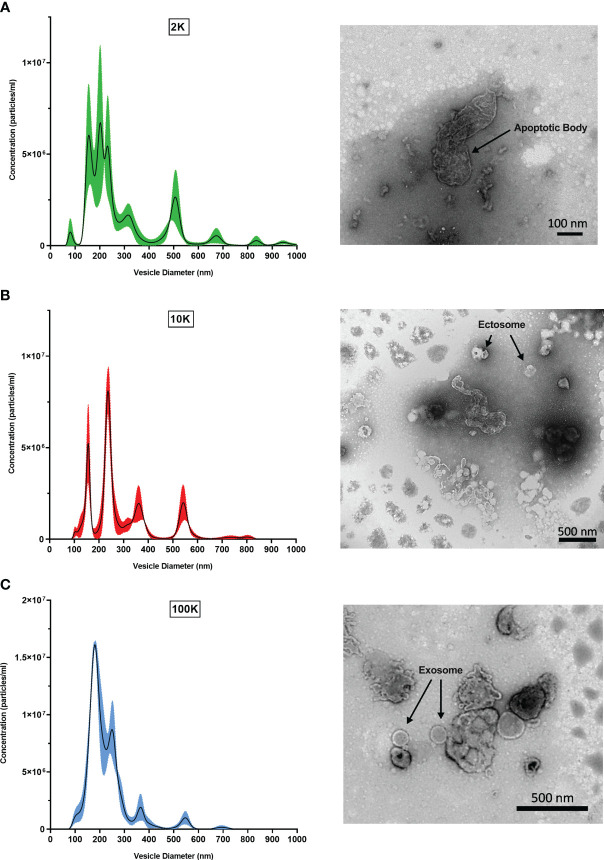
Fractions of *Leishmania major*-derived extracellular vesicles display distinct morphologic characteristics. Fractionated EV populations derived from *Leishmania major* are analyzed by Nanoparticle Tracking Analysis (NTA) to determine diameter distributions of particles present in the **(A)** 2K, **(B)** 10K, and **(C)** 100K fractions (left). Corresponding Transmission Electron Microscopy (TEM) images (right) show representative EV morphology within each fraction. Bar, 100nm; Magnification, 30000x; or Bar, 500nm; Magnification, 9300x.

To first validate that the fractions corresponded to their respective classes of *Leishmania* spp.-derived EVs, we performed TEM and NTA to observe distinct morphological characteristics. NTA revealed that the three preparations were composed of EVs with different size distributions, peaks, and curve profiles (**Figure 1**). The 2K fraction displayed the highest EV diameters, followed by the 10K fraction and the 100K fraction. Furthermore, the 100K fraction contained the highest concentration of vesicles with a diameter from 100-300 nm, indicating an enrichment in small EVs. TEM observations were consistent with the NTA results, revealing that the 2K fraction contained a diverse and heterogenous population of vesicles with large diameters, and that the 10K fraction consisted of intermediate-sized vesicles. The 100K fraction was composed of small EVs, including those with a diameter of 50-150 nm and a cup-shaped double membrane, which are qualities characteristic of exosomes (**Figure 1**) (Hosseini et al., 2013). Together, these data indicate that the 2K, 10K, and 100K extracted fractions are composed of EVs that display characteristic morphological traits of large vesicles (e.g., apoptotic bodies), intermediate-sized vesicles (e.g., ectosomes), and small vesicles (e.g., exosomes), respectively.

### Fractionated leishmanial extracellular vesicle populations display distinct proteome functionality and expression profiles

**Figure 2:**
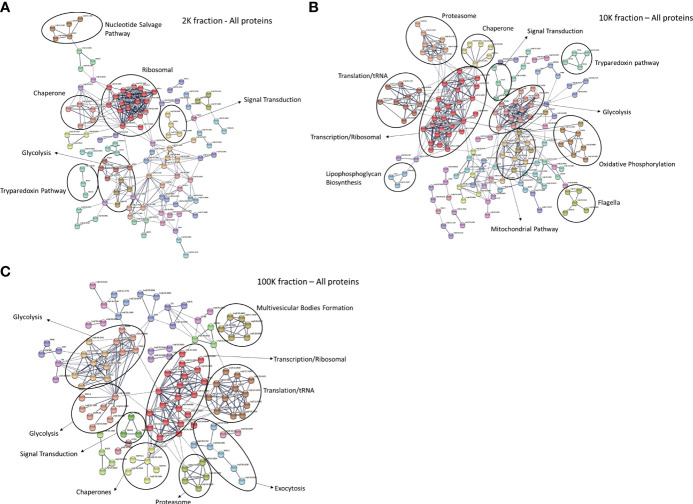
Protein-protein interaction networks of total proteins detected in fractions of *Leishmania major*-derived extracellular vesicles display notable variations. Networks were generated using total proteins detected by LC-MS/MS in **(A)** 2K, **(B)** 10K, and **(C)** 100K fractions of *Leishmania major-*derived EVs. In the 2K/apoptotic body fraction **(A)**, notable clusters include the Nucleotide Salvage Pathway, Ribosomal, Chaperone, Signal transduction, Glycolysis and the Tryparedoxin pathway. In the 10K/Ectosome fraction **(B)**, clustering patterns include Proteasome, Chaperone, Tryparedoxin pathway, Translation/tRNA, Ribosome, Signal transduction, Glycolysis, Lipophosphoglycan biosynthesis, Mitochondria, Oxidative phosphorylation, and Flagella. In the 100K/exosome fraction **(C)** clusters include Glycolysis, Signal transduction, Chaperone, Proteasome, Translation/tRNA, Ribosome, along with unique clusters representing Multivesicular Body Formation and Exocytosis. Protein-protein interaction networks of the identified proteins were created using String database (https://www.string-db.org) using the highest confidence of interaction (>0.900), showing only connected proteins in the network and using an MCL inflation parameter of 3.

Following the confirmation of distinct morphological traits of the EVs present in each fraction, we conducted a proteomic analysis of the three fractions using LC-MS/MS and a bioinformatic workflow. When mapped to the *L. major* database, our analysis identified 610 proteins in the 2K fraction, 754 proteins in the 10K fraction, and 568 proteins in the 100K fraction (**Data Sheet 1**). Of these, the 100K fraction, which contained the highest proportion of small EVs, displayed the highest relative abundance of canonical exosomal markers (**Supplementary Figure 1**). To further characterize the proteomic landscape of each fraction, we constructed protein-protein interaction (PPI) networks of the proteins identified in each EV subgroup using StringDB. The PPI network for the 2K fraction revealed large clusters associated with ribosomal functions, chaperones, and the nucleotide salvage pathways (**Figure 2A**). The 10K network had significant clustering associated with chaperones, signal transduction, tryparedoxin, glycolysis, ribosomal functions, mitochondrial functions, flagellar functions, oxidative phosphorylation, and translation/tRNA pathways (**Figure 2B**). The PPI network for the 100K fraction displayed clusters associated with glycolysis, ribosomal functions, chaperones, and translation/tRNA pathways, as well as unique clusters related to exocytosis and multivesicular body formation (**Figure 2C**). Overall, these networks revealed common and distinct functionalities between the proteomes of the different fractions, such as the mitochondrial pathways in the 10K fraction and exocytosis/MVB Formation in the 100K fraction.

**Figure 3:**
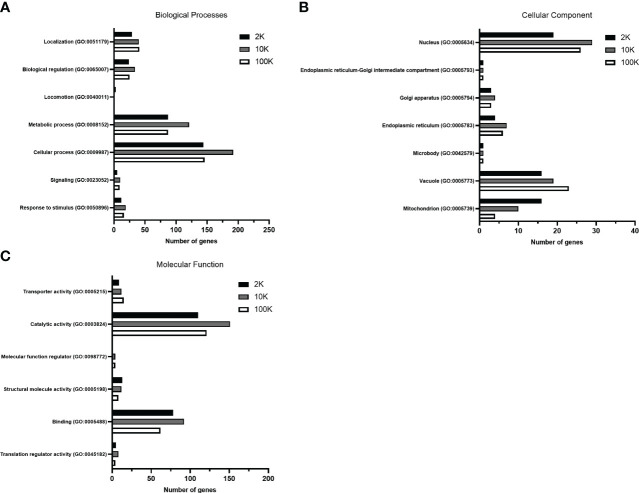
Gene Ontology analysis of total proteins detected in fractions of *Leishmania major*-derived extracellular vesicles display notable variations. Proteins detected by LC-MS/MS in 2K, 10K, and 100K fractions of *Leishmania major-*derived EVs were annotated using PANTHER (pantherdb.org) gene ontology analysis (GO) according to **(A)** Biological Processes, **(B)** Molecular Function, and **(C)** Cellular Component (Level 3 – membrane-bound organelle). When classified according to biological processes and molecular functions, no significant changes are apparent in terms of percentage of the total classified proteins. Most significant differences are regarding the cellular component, suggesting that the cellular origins of the EV fractions are **(A)** mitochondrial, **(B)** nuclear and **(C)** vacuolar/endosomal.

To further establish functional differences between the proteomes of each EV fraction, we analyzed the total detected proteins using the PANTHER database for gene ontology – a bioinformatic analysis workflow which classifies and identifies gene products’ functions based on their family and subfamily, molecular function, biological processes, and cellular components. Of note, no significant differences in the percentages of total classified proteins for each subcategory were apparent between the fractions when classified according to biological process and molecular function, despite the total number of identified proteins varying between the samples (**Supplementary Tables 1, 2**) (**Figures 3A, C**). However, when classified according to cellular components, notable differences between the fractions were observed, suggesting differences in cellular origin between the EV populations (Level 3 - Membrane-bound organelle) (**Supplementary Table 3**) (**Figure 3B**). The percentage of total protein composition attributed to a mitochondrial origin showed an evident decreasing pattern in the 2K, 10K, and 100K fractions, which were 29.60%, 15.60%, and 7%, respectively, while proteins of vacuolar origin increased from 29.60% to 30.20% and 40.40%, respectively (**Supplementary Table 3**). We then delved deeper into the proteins of vacuolar origin in each fraction, which are involved in EV biogenesis, and identified several proteins related to the ESCRT pathway in the 100K fraction, notably CHMP2A, CHMP2B, SNF-7, and VTA1. Analysis of the proteomic landscapes of each EV fraction using the PANTHER database revealed their differential cellular origins and underscored a remarkable enrichment of proteins involved in mitochondrial function, vacuolar sorting and the ESCRT pathway in the 100K fractionated EV population. Overall, this analysis indicated that the three fractions comprised different functional elements of varying cellular origins.

**Figure 4:**
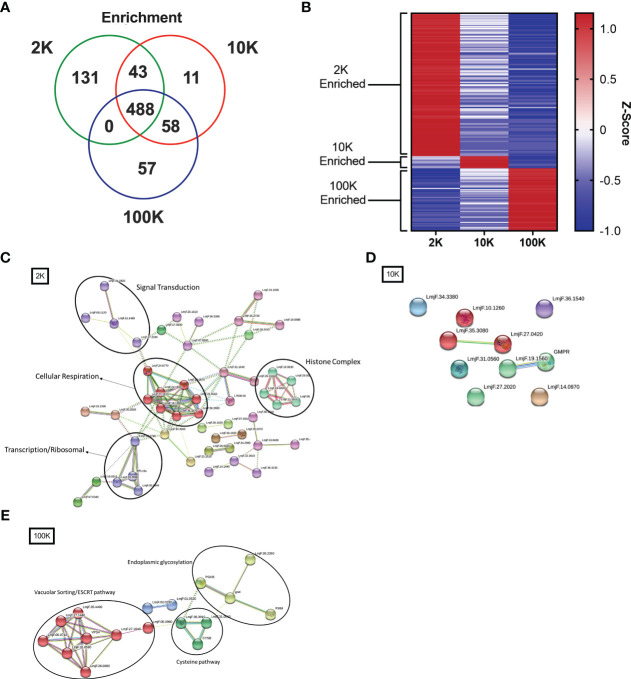
Fractions of *Leishmania major*-derived extracellular vesicles display distinct quantitative proteomic profiles and functionality. The protein content of *Leishmania major-*derived EVs was catalogued by mass spectrometry (*L. major* database) using biological triplicates obtained for each fraction (*n=3*) and analyzed using Mascot followed by Scaffold Software. Quantitative profiles are represented both in absolute expression using **(A)** a Venn diagram, and in relative expression using **(B)** a heat map. Enrichment of proteins detected by LC-MS/MS was determined using ANOVA without corrections (p<0.05) on pre-established true-hit proteins. Enrichment was defined as expression levels greater than 2 standard deviations from the mean of all LC-MS/MS samples. The heat map was generated using Z-scores (calculation provided in materials and methods) without normalization, clustered into groups based on protein sets exhibiting positive Z-scores in each group. Protein-protein interaction networks of enriched proteins were generated using enriched proteins detected by LC-MS/MS in **(C)** 2K, **(D)** 10K, and **(E)** 100K fractions of *Leishmania major-*derived EVs. Notable protein clusters for each fraction included: **(C)** Histone Complex, Signal Transduction, Transcription/Ribosomal, and Cellular Respiration in the 2K fraction; **(D)** no notable clusters in the 10K fraction; and **(E)** clusters associated to Endoplasmic Glycosylation, Cysteine Pathway, and Vacuolar Sorting/ESCRT pathway in the 100K fraction. Protein-protein interaction networks of the identified proteins were created using String database (https://www.string-db.org) using the highest confidence of interaction (>0.900), showing only connected proteins in the network and using an MCL inflation parameter of 3.

To further investigate the differences between the proteomes of each EV fraction, we conducted a quantitative analysis of the detected proteins to determine their expression levels and enrichment in EV subpopulations. Our analysis revealed that there were 131 proteins significantly enriched in the 2K fraction, 11 in the 10K fraction, and 57 in the 100K fraction, and that these enriched protein sets shared no overlap between fractions (**Figures 4A, B**; **Table 1**) (**Data Sheet 2**). These data further corroborated that these fractions are composed of different EV populations and confirmed the presence of enriched protein sets within each class of *Leishmania* spp.-derived EVs.

**Table 1:** Enriched proteins identified in 2K, 10K and 100K EV fractions.

Enriched Fraction	Protein name	Accession Number (UniProt)	Molecular Weight
2K	Tubulin alpha chain	E9AGK7_LEIIN	54 kDa
	Paraflagellar rod protein 1D	E9AE36_LEIMA	69 kDa
	ATP-dependent RNA helicase	A4I7K4_LEIIN	67 kDa
	Tubulin beta chain	F8QV42_LEIDO	50 kDa
	Proline oxidase, mitochondrial-like protein	Q4Q933_LEIMA	64 kDa
	Dynein heavy chain	Q4Q9Z6_LEIMA	535 kDa
	Tubulin beta chain	Q4QI42_LEIMA	50 kDa
	Succinate dehydrogenase [ubiquinone] flavoprotein subunit, mitochondrial	Q4QAG8_LEIMA	67 kDa
	Dynein heavy chain	Q4QFY9_LEIMA	529 kDa
	Pyruvate dehydrogenase E1 component alpha subunit	Q4QDQ1_LEIMA	43 kDa
	4-coumarate:coa ligase-like protein	Q4QDB5_LEIMA	66 kDa
	ATP-dependent DEAD-box RNA helicase	E9AEL4_LEIMA	46 kDa
	Voltage-dependent anion-selective channel	E9ACB9_LEIMA	30 kDa
	Thiolase protein-like protein	Q4Q698_LEIMA	47 kDa
	2-oxoglutarate dehydrogenase E1 component	Q4Q171_LEIMA	111 kDa
	Succinate–coa ligase [ADP-forming] subunit alpha, mitochondrial	A4I1R2_LEIIN	31 kDa
	Malic enzyme	Q4QAQ6_LEIMA	63 kDa
	Succinyl-coa:3-ketoacid-coenzyme A transferase	Q4Q3V3_LEIMA	53 kDa
	2-methoxy-6-polyprenyl-1,4-benzoquinol methylase, mitochondrial	Q4Q0M6_LEIMA	32 kDa
	Acyl-coa dehydrogenase	Q4Q812_LEIMA	69 kDa
	Possible 3-ketoacyl-coa thiolase	Q9U0V9_LEIMA	47 kDa
	Calpain-like cysteine peptidase	E9AD26_LEIMA	521 kDa
	Polyadenylate-binding protein	A4IBV3_LEIIN	65 kDa
	60S ribosomal protein l10a	Q4QDX9_LEIMA	25 kDa
	Beta tubulin (Fragment)	Q1PCJ8_NEODS	13 kDa
	RNA binding protein	Q4Q5K7_LEIMA	25 kDa
	60S ribosomal protein L9	Q4Q6V5_LEIMA	22 kDa
	40S ribosomal protein S15A	A4HV26_LEIIN	15 kDa
	Elongation factor Tu	Q4QDW8_LEIMA	52 kDa
	Axoneme central apparatus protein	E9AUS9_LEIMU	55 kDa
	ATP synthase, epsilon chain	Q4Q6S8_LEIMA	20 kDa
	Paraflagellar rod component par4	Q4QJJ9_LEIMA	68 kDa
	ATP-dependent Clp protease subunit, heat shock protein 78 (HSP78), putative	E9B7L5_LEIDB	91 kDa
	Kinetoplast-associated protein-like protein	E9AD01_LEIMA	79 kDa
	40S ribosomal protein S16	Q4Q9A5_LEIMA	17 kDa
	40S ribosomal protein S19 protein	A4IA55_LEIIN	20 kDa
	Trifunctional enzyme alpha subunit, mitochondrial-like protein	Q4Q939_LEIMA	79 kDa
	Leucine-rich repeat protein	Q4QHX1_LEIMA	106 kDa
	Dihydrolipoamide acetyltransferase component of pyruvate dehydrogenase complex	Q4Q1F5_LEIMA	49 kDa
	Phosphodiesterase	Q6S997_LEIMA	103 kDa
	Nucleolar protein	Q4QHJ7_LEIMA	53 kDa
	V-type proton atpase subunit a	Q4QAY7_LEIMA	88 kDa
	Histone H4	Q4QJ78_LEIMA	11 kDa
	Histone H2B	Q4QDL6_LEIMA	12 kDa
	Transcription factor-like protein	E9ADK4_LEIMA	115 kDa
	Dynein heavy chain	Q4Q992_LEIMA	458 kDa
	Heat shock protein	Q4Q3U8_LEIMA	72 kDa
	Dynein heavy chain	Q4Q2F4_LEIMA	529 kDa
	RNA helicase	A4HZF8_LEIIN	59 kDa
	Glycoprotein 96-92	Q4Q843_LEIMA	87 kDa
	Regulatory subunit of protein kinase a-like protein	Q4Q2R2_LEIMA	72 kDa
	Paraflagellar rod component	Q4QHP3_LEIMA	68 kDa
	ATP-dependent DEAD/H RNA helicase	Q4QIQ9_LEIMA	64 kDa
	Histone H2A	Q4QCA4_LEIMA	14 kDa
	Transmembrane 9 superfamily member	Q4Q2H8_LEIMA	71 kDa
	Splicing factor ptsr1-like protein	Q4QIK0_LEIMA	42 kDa
	Dynein heavy chain	Q4Q8K9_LEIMA	478 kDa
	Dihydrolipoamide acetyltransferase	Q4QCG0_LEIMA	40 kDa
	Nucleolar RNA-binding protein	A4HTC3_LEIIN	22 kDa
	Phosphodiesterase	Q6S996_LEIMA	104 kDa
	Dynein heavy chain	Q4Q7X4_LEIMA	474 kDa
	Flagellar radial spoke protein	Q4QGB1_LEIMA	66 kDa
	Isovaleryl-coa dehydrogenase	E9AD70_LEIMA	45 kDa
	Aromatic amino acid hydroxylase-like	Q6WRI4_LEIMA	51 kDa
	Poly-zinc finger protein 2	Q4Q1R1_LEIMA	15 kDa
	Chaperonin HSP60/CNP60	Q4Q711_LEIMA	58 kDa
	4-coumarate:coa ligase-like protein	Q4QDB7_LEIMA	66 kDa
	Branched-chain amino acid aminotransferase	E9ADI3_LEIMA	44 kDa
	Dynein	Q4Q5H6_LEIMA	68 kDa
	Acyl-coa dehydrogenase	E9AF97_LEIMA	45 kDa
	Histone H3 variant	Q4QDF8_LEIMA	16 kDa
	Electron transfer flavoprotein-ubiquinone oxidoreductase	Q4QIN2_LEIMA	63 kDa
	Mitochondrial RNA binding protein	Q4QB87_LEIMA	40 kDa
	Heat shock protein-like protein	Q4Q584_LEIMA	36 kDa
	Histone H3	H3_LEIIN	15 kDa
	3-hydroxy-3-methylglutaryl coenzyme A reductase	A4I602_LEIIN	46 kDa
	Beta tubulin (Fragment)	Q1PCK0_CRIFA	14 kDa
	Beta-tubulin	A4HLD6_LEIBR	21 kDa
	RNA-binding protein	E9AHW1_LEIIN	30 kDa
	Citrate synthase	Q4QDX2_LEIMA	50 kDa
10K	Inosine-5’-monophosphate dehydrogenase	Q4QD53_LEIMA	56 kDa
	GMP reductase	GMPR_LEIMA	52 kDa
	Glycerol kinase, glycosomal	E9AFD2_LEIMA	56 kDa
	Mevalonate kinase	Q4Q6K7_LEIMA	35 kDa
	D-lactate dehydrogenase-like protein	E9ADI2_LEIMA	54 kDa
	Arginase	A0A145YEM0_LEIMA	36 kDa
	Ribokinase	RBSK_LEIMA	35 kDa
	Aldose 1-epimerase-like protein	Q4Q2K6_LEIMA	33 kDa
100K	Plasma membrane atpase	Q4QDN7_LEIMA	107 kDa
	Long chain fatty Acyl coa synthetase	E9ACH0_LEIMA	79 kDa
	Adenosylhomocysteinase	Q4Q124_LEIMA	48 kDa
	Phosphoglycerate kinase	Q4QD33_LEIMA	45 kDa
	Glucose transporter, lmgt2	Q4Q0D1_LEIMA	61 kDa
	Long-chain-fatty-acid-coa ligase	E9AC43_LEIMA	78 kDa
	Thimet oligopeptidase	Q4Q937_LEIMA	77 kDa
	Aminopeptidase P	E9AF59_LEIMA	54 kDa
	Inosine-guanosine transporter	Q4Q1M9_LEIMA	54 kDa
	5-methyltetrahydropteroyltriglutamate-homocystein e S-methyltransferase	Q4Q6R3_LEIMA	86 kDa
	Calcium motive p-type atpase	E9AF31_LEIMA	122 kDa
	Vacuolar protein sorting-associated protein 4	E9AEB2_LEIMA	50 kDa
	Metallo-peptidase, Clan MA(E), Family M32	Q4Q3T3_LEIMA	57 kDa
	60S ribosomal protein L17, putative	A0A088RXP1_9TRYP	19 kDa
	6-phosphogluconate dehydrogenase, decarboxylating (Fragment)	Q9NGR0_LEIMA	52 kDa
	Ef-hand protein 5	A4HU12_LEIIN	21 kDa
	Phospholipid-transporting atpase	Q4QG01_LEIMA	124 kDa
	Dipeptidyl peptidase 3	Q4QJA6_LEIMA	76 kDa
	Pyridoxal kinase, putative	E9BLM4_LEIDB	33 kDa
	Amino acid permease	Q4Q682_LEIMA	64 kDa
	Cystathionine beta-synthase	Q4QEG9_LEIMA	39 kDa
	Phosphomannomutase	Q4Q1M7_LEIMA	28 kDa
	Dipeptylcarboxypeptidase	E9ACE8_LEIMA	77 kDa
	Peptidase M20/M25/M40	Q4Q426_LEIMA	52 kDa
	ATP-binding cassette protein subfamily A,member 8	E9AD74_LEIMA	202 kDa
	Amino acid permease	Q4QBX3_LEIMA	54 kDa
	Conserved SNF-7-like protein	E9AEN3_LEIMA	26 kDa
	Surface antigen protein	Q4QGL8_LEIMA	65 kDa
	Sucrose-phosphate synthase-like protein	Q4QES5_LEIMA	52 kDa
	Folate/biopterin transporter	Q4QHH9_LEIMA	77 kDa
	Arginyl-trna synthetase	E9ADB2_LEIMA	78 kDa
	Kinesin-like protein	A4HY48_LEIIN	94 kDa
	Amino acid transporter aatp11	Q4Q6M9_LEIMA	52 kDa

Differential expression was determined using Scaffold Software, with enrichment defined as peptide counts greater than 2 standard deviations from the experiment mean (p ≤ 0.05, ANOVA). Uncharacterized proteins have been excluded from this table for clarity (see **Data Sheet 2** for exhaustive list).

To further characterize the functionality of such enriched proteins, we used StringDB PPI analysis on the enriched protein sets to generate networks of functional and physical interaction clusters. The network of proteins enriched in the 2K fraction displayed four distinct clusters related to histone complexes, signal transduction, transcription/ribosomal pathways, and cellular respiration. In contrast, no significant interaction networks could be identified for the 10K fraction, likely due to few enriched proteins identified within the fraction (**Figure 4D**). Enriched proteins in the 100K fraction formed three main clusters: endoplasmic glycosylation, cysteine pathway, and vacuolar sorting/ESCRT pathway (**Figures 4C, E**). These data highlight that the proteins enriched in each EV fraction have distinct functional and biological roles, confirming the distinction between the fractionated populations.

We then compared the quantitative protein expression profiles between the three distinct fractions by generating volcano plots (2K vs. 100K, 2K vs. 10K, and 10K vs. 100K), plotting the fold change of protein expression against the significance of enrichment. Each plot showed a distinct protein expression spread, identifying proteins significantly enriched in each EV fraction. For example, when comparing the 2K and 100K fractions, proteins related to aerobic metabolism, notably citrate synthase and ATP synthase, were significantly enriched within the 2K fraction, whereas important parasitic virulence factors, like EF-1, GP63, and superoxide dismutase, were enriched within the 100K fraction (**Supplementary Figure 2**; **Data Sheet 3**). Furthermore, this same enrichment of parasitic virulence factors in the 100K fraction was observed in comparison to the 10K fraction, with additional enrichment of VPS4, an important component of the ESCRT complex. Additionally, this analysis identified cellular metabolic pathway-related proteins enriched within the 10K fraction as compared to the 100K fraction, particularly enzymes related to glycolysis and the citrate cycle, such as glyceraldehyde 3-phosphate dehydrogenase, ATP-dependent 6-phosphofructokinase, and citrate synthase (**Supplementary Figure 2**; **Data Sheet 3**). Finally, when comparing the 2K and 10K fraction proteomes, enrichment of citrate cycle proteins, such as citrate synthase and isocitrate dehydrogenase, was observed in the 2K fraction, while the 10K fraction displayed enrichment of glycolysis-related proteins like enolase and pyruvate kinase (**Supplementary Figure 2**; **Data Sheet 3**). In summary, this analysis confirmed our previous observations that each EV fraction has distinct characteristics of protein enrichment and functionality, particularly in cellular aerobic metabolism in both the 2K and 10K fractions and virulence factors and ESCRT pathway proteins in the 100K fraction.

### Classical biomarkers for leishmanial exosome validation are non-specific to small extracellular vesicles

To gain further insight into the characteristics of the *Leishmania* spp.-derived exoproteome, we conducted a detailed analysis of the key proteins identified, comparing them with established predictive markers such as HSP70, HSP83, GP63, Tryparedoxin peroxidase, Enolase, EF-1, and Surface Antigen Protein, among others. Both HSP83 and HSP70 were identified in all three EV fractions, with similar expression levels. In fact, while HSP70 was slightly upregulated in the 10K fraction, this was not statistically significant. In addition, there was no significant variation in the expression levels of EF-1, Tryparedoxin peroxidase, Calpain-like cysteine peptidase, and 14-3-3 protein between the fractions. GP63, Enolase, and Surface Antigen Protein did, however, show an increasing trend in expression from the 2K to the 100K fraction (**Supplementary Table 4**). Together, these findings suggest that common predictive markers for *Leishmania* spp.*-*derived exosomes are non-specific, though key virulence factors such as GP63 are significantly enriched in populations of smaller leishmanial EVs.

### Proteins involved in the leishmanial ESCRT pathway and the phosphatase system are specific to *Leishmania* spp.-derived exosomes

**Figure 5:**
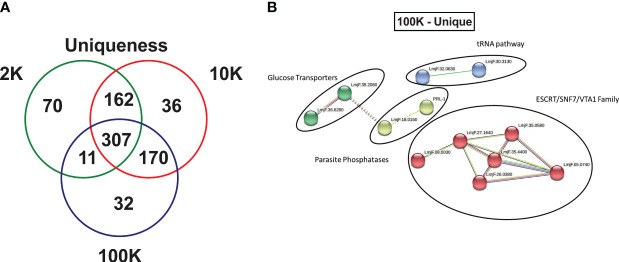
Fractions of *Leishmania major*-derived extracellular vesicles display distinct uniquely expressed proteins. **(A)** The protein content of *Leishmania major-*derived EVs was catalogued by mass spectrometry (*L. major* database) using biological triplicates obtained for each fraction (*n=3*) and analyzed using Mascot followed by Scaffold Software. Total proteins detected by LC-MS/MS in each fraction were determined using inclusion criteria of a minimum of 2 detected peptides, a peptide threshold of 80%, and a protein identity of 95%. Uniqueness was then determined based on a true hit being identified in only one of the fractions. **(B)** A protein-protein interaction networks of uniquely expressed proteins in the 100K fraction was generated using unique proteins detected by LC-MS/MS in the 100K fraction of *Leishmania major-*derived EVs. Notable proteins include SNF-7, Qc-SNARE, IST1, SNF-7 superfamily, VTA1, CHMP2B, ALIX, and VPS37 were identified in this group, which are part of the ESCRT pathway, and PP5 and PRL-1 which are parasite phosphatases were also identified. The protein-protein interaction network was created using String database (https://www.string-db.org) using the highest confidence of interaction (>0.900), showing only connected proteins in the network and using an MCL inflation parameter of 3.

Finally, an in-depth investigation was conducted to identify potential novel markers of *Leishmania* spp.-derived exosomes. The study focused on the 70, 36, and 32 proteins that were uniquely identified in the 2K, 10K, and 100K EV fractions, respectively (**Figure 5A**; **Supplementary Tables 5–7**). Notably, the 100K fraction exhibited the presence of SNF-7 and Qc-SNARE proteins, which are both involved in the ESCRT pathway, suggesting their potential as identifying markers. Additionally, 14 proteins were identified in the 100K fraction that were uncharacterized in the UNIPROT database, necessitating further examination. Homology searches conducted on closely related species in UNIPROT allowed for the identification of six homologous proteins involved in the ESCRT pathway: IST1, SNF-7 superfamily, VTA1, CHMP2B, ALIX, and VPS37 (**Supplementary Table 7**).

To further characterize the 32 proteins that were uniquely expressed in the 100K fraction, StringDB analysis was conducted, revealing four protein-protein interaction clusters which could be classified as ESCRT pathway-related (SNF-7 and Qc-SNARE), tRNA-associated, Glucose Transporters, and Parasite Phosphatases (PP5 and PRL-1) (**Figure 5B**).

## Discussion

*Leishmania* spp. utilize parasitic virulence factors such as GP63, LPG-1, and EF-1 to drive immunopathology in their mammalian hosts by acting on host immune cells and abrogating leishmanicidal functions (Olivier et al., 2012; Späth et al., 2000; Nandan et al., 2002; Silverman et al., 2010b). Over the last decade, several research groups have studied the role of leishmanial exosomes in immunopathogenesis, though the lack of specific markers for these extracellular vesicles remains a major challenge in the field (Silverman et al., 2010a; Silverman and Reiner, 2012; Santarém et al., 2013; Atayde et al., 2015).

For our study, we used serial differential ultracentrifugation as previously described by Kowal et al. to isolate three distinct populations of *Leishmania* spp.*-*derived extracellular vesicles – 2K, 10K and 100K fractions – corresponding to large vesicles (e.g., apoptotic bodies), intermediate-sized vesicles (e.g., ectosomes), and small vesicles (e.g., exosomes) (Kowal et al., 2016). While this technique results in the recovery of distinct EV populations with different size distributions, for which the major component is the vesicle class of interest, cross-contamination between fractions is possible due to the heterogenous EV sizes.

Data collected using both TEM and NTA suggested that each fraction contained a distinct extracellular vesicle population, and that the fraction containing small vesicles/exosomes (100K) was composed of relatively homogeneous EVs with a smaller diameter than that observed in other fractions (**Figure 1**). Direct (TEM) and indirect (NTA) morphological analysis confirmed this, validating our isolation and fractionation methods. These results are in agreement with Kowal et al.’s work on dendritic cell-derived EVs, whereby homogenous EV populations with distinct morphological characteristics were recovered in each fraction, though they recovered larger populations of small EVs (<200 nm) (Kowal et al., 2016). This is likely due to the methodology underlying leishmanial exosome isolation, as a temperature shift is used to stimulate EV production, inducing the packaging of heat shock proteins and large stress-induced cargo (Hassani et al., 2011; Kowal et al., 2016; Vucetic et al., 2020). Further, as distributions are similar but slightly shifted towards larger EVs, this finding is a potential artifact of the use of different nanoparticle tracking analysis machinery.

LC-MS/MS proteomic analysis and downstream bioinformatic mapping of the fractionated EV populations further corroborated the divergence of EV subpopulations in each fraction. Functional characterization with protein-protein interaction analysis (StringDB) indicated that the proteins detected in EV subpopulations had distinct functionality. While mitochondrial proteins formed an important cluster in the 10K fraction, exocytosis and MVB formation associated proteins were unique to the 100K fraction – an observation which validates the origin of the small extracellular vesicles, as these functions are directly related to known pathways for the intracellular formation and outtake of exosomes by eukaryotic cells (Piper and Katzmann, 2007). However, mitochondrial-related proteins would have been expected in the 2K fraction rather than the 10K fraction, considering apoptotic bodies use mitochondrial pathways for their formation. This finding is likely due to the overlap of intermediate and large-sized vesicles during the isolation process, with potential precipitation of larger vesicles within the 10K fractions or co-precipitation of free mitochondrial fragments along with this fraction. Though this highlights a potential technical limitation of EV isolation, Gene Ontology Analysis (PANTHER-db) revealed an overall decrease of proteins of mitochondrial origin from the 2K fraction to the 10K fraction, indicating that the 2K fraction is the most enriched in apoptotic bodies. Further, proteins of vacuolar origin are most abundant in the 100K fraction, suggesting the presence of exosomes, as their biogenesis requires the formation of vacuoles within multivesicular bodies using the ESCRT pathway. Several proteins involved in this pathway, such as CHMP2A, CHMP2B, SNF-7, and VTA1, which are essential to this process, are uniquely detected within the 100K fractions, validating its composition in small extracellular vesicles with exosome characteristics (Hanson et al., 2009).

Moreover, quantitative analysis of the proteomes of each fraction enabled the identification of protein sets that were common and enriched in one fraction of EVs or the other. Of notable interest is the inverse relationship between upregulated and downregulated proteins in the 2K and 100K fractions, as visualized heat map, indicating minimal overlap between these protein sets. This not only confirms that minimal contamination by the other class of EVs occurred in the extremum fractions, but also highlights the vastly different roles these EVs play. Further, moderate overlap of the 10K fraction with both the 2K and 10K fraction suggests the presence of low numbers of other EV populations, though a clearly enriched set of proteins indicates relative purity of the preparation. PPI analysis of enriched protein sets permitted the identification of clusters related to the Histone Complex, Signal Transduction, Transcription/Ribosomal, and Cellular Respiration in the 2K complex. In contrast, clusters related to Endoplasmic Glycosylation, the Cysteine Pathway, and the Vacuolar Sorting/ESCRT pathway were identified in the 100K fraction (**Figures 4C, E**). This observation reiterates the importance of the ESCRT pathway in exosome formation within the cell – a pathway that has been experimentally demonstrated by several research groups (Saksena et al., 2007; Williams and Urbé, 2007; Hanson et al., 2009). Although the association between exosomes and both the endoplasmic glycosylation and cysteine pathways is less clear, these clusters may be due to the importance of the ER and the Golgi apparatus in cargo sorting before packaging and trafficking into exosomes (Xiang et al., 2013). Further studies are necessary to elucidate a clear link between these two processes and the eukaryotic cell production of small extracellular vesicles.

Though minimal overlap exists between the proteomes of leishmanial EVs and that identified in Kowal et al.’s study of mammalian exosomes, largely due to the absence of homologous proteins across the studied organisms, the overall functionality of the proteomes varies across EV populations in a similar way, as visualized by the step-wise decrease in mitochondrial proteins as EVs decrease in size (Kowal et al., 2016).

On the other hand, the volcano plots used to quantitatively analyze protein sets provided evidence of differential protein expression levels among the fractions. The 100K fraction was found to be enriched with key parasitic virulence factors, such as EF-1, GP63, and superoxide dismutase, when compared to both the 2K and 10K fractions. This finding is consistent with previous studies on *Leishmania* spp.-derived exosomes (Hassani et al., 2011; Atayde et al., 2015). Furthermore, glycolysis-related proteins, including enolase and pyruvate kinase, were found to be enriched in both the 2K and 10K fractions, suggesting the presence of EVs associated with aerobic metabolism, such as apoptotic bodies and ectosomes. Thus, it can be concluded that the 100K fraction contains a unique population of EVs, likely of exosomal origin.

Similarly, recent studies on *Leishmania* spp.-derived exosomes have used markers such as HSP70 and HSP83 and the parasitic virulence factors GP63 and EF-1 to identify this group of EVs (Silverman et al., 2010b; Hassani et al., 2011; Atayde et al., 2015). However, both HSP70 and HSP83 were detected in all three EV fractions, indicating that these markers are non-specific. While serial differential ultracentrifugation does not permit the recovery of completely homogenous EV fractions, should this non-specificity be a result of exosomal contamination in other EV fractions, lower abundance of these canonical markers would be expected in larger EV fractions. Thus, the similar abundance of HSP70 and HSP83 across EV populations suggests that this is not an artifact of contamination, but rather the result of the proteins being packaged in all EVs. This finding mirrors data published by Kowal et al., whereby canonical markers of mammalian exosomes are similarly non-specific to small extracellular vesicles (Kowal et al., 2016).

Levels of GP63, Enolase, and Surface Antigen Protein increased throughout the fractions, with the highest concentration found in the 100K fraction, further suggesting that these EVs are exosomes, which tend which tend to accumulate high concentrations of parasitic virulence factors. Notably, the enrichment in GP63 suggests that the parasite actively uses these vesicles to deliver parasitic proteins into mammalian host immune cells, which have immunomodulatory effects (Atayde et al., 2015). The abundance of GP63 has also been shown to be highest in exosomes produced by infective metacyclic- and stationary-stage parasites, while exhibiting much lower levels in logarithmic-stage parasites – a finding that further implicates exosome-secreted GP63 in the immunopathogenesis of leishmaniasis (Marshall et al., 2018). Together, this suggests differential expression of GP63 in a life cycle-dependent manner, and intentional sorting of the surface metalloprotease into exosomal cargo. Additional studies addressing the content of all extracellular vesicle classes at different life stages may provide more insight into this process.

Furthermore, several proteins, including SNF-7, Qc-SNARE, IST1, SNF-7 superfamily, CHMP2B, ALIX, and VPS37, were detected exclusively in the 100K fraction. These proteins play a role in the ESCRT pathway, which is responsible for the endosomal formation and trafficking of small extracellular vesicles, including exosomes (Piper and Katzmann, 2007; Hanson et al., 2009). Although these proteins are relatively conserved between species, they have specificity for small extracellular vesicles. Thus, they may be effective markers for identifying *Leishmania* spp.*-*derived exosomes, despite the limitations of identifying exosomes of leishmanial origin in a heterogenous mixture of vesicles from different organisms.

Other proteins identified in this study include parasitic phosphatases such as PP5 and PRL-1, which have previously been identified as virulence factors secreted by *Leishmania* spp. via the exosomal pathway (Leitherer et al., 2017). PRL-1 is a *Leishmania*-specific protein, and was uniquely detected in the 100K fraction, indicating that it could be used as a marker for identifying leishmanial exosomes. In fact, all PRL-1 homologous proteins indexed in UniProt’s database are expressed exclusively by species of *Leishmania*, minimizing the risk of cross-identification with other organisms.

In summary, our study utilized a comprehensive bioinformatic analysis of small EVs collected through serial differential ultracentrifugation, which was further validated to identify potential proteomic markers of *Leishmania* spp.-derived exosomes. These include ESCRT proteins SNF-7, Qc-SNARE, IST1, VTA1, CHMP2B, ALIX, and VPS37, as well as the parasitic phosphatase PRL-1. While ESCRT proteins may be conserved between organisms, PRL-1 shows promise as an organism-specific exosome marker and requires further investigation. Further studies are necessary to assess whether these markers are shared by other *Leishmania*, including species causing visceral and mucocutaneous infection.

**Figure 6:**
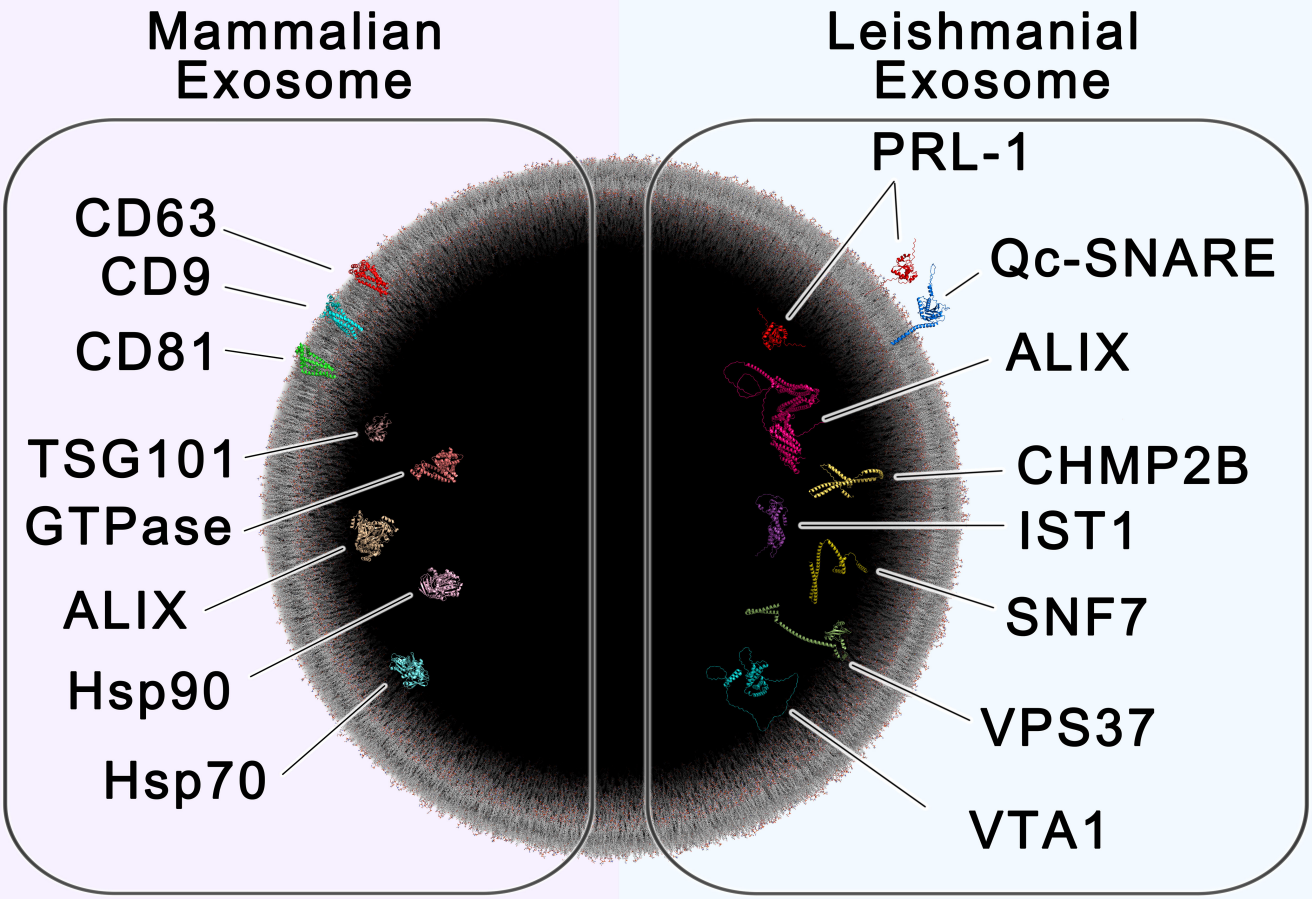
– All-Atom Scale Model of Mammalian and Leishmanial Exosomes. The cross section of a 100 nm extracellular vesicle is shown, containing protein markers of mammalian exosomes (left) and leishmanial exosomes (right). Established biomarkers of mammalian exosomes include the tetraspanins CD63, CD9 and CD81, the chaperones HSP70 and HSP90, tumor susceptibility gene 101 (TSG101), GTPase, and ALIX (Shaabani et al., 2023). Proteins found to be enriched in leishmanial exosomes include the ESCRT proteins Vacuolar-sorting protein SNF7 (SNF-7), Qc soluble N-ethylmaleimide-sensitive factor-attachment protein receptor (Qc-SNARE), Vacuolar protein sorting-associated protein IST1 (IST1), Vacuolar protein sorting-associated protein VTA1 (VTA1), Charged multivesicular body protein 2b (CHMP2B), Vacuolar-sorting protein BRO1 (ALIX), and Vacuolar-sorting protein 37 (VPS37), which are involved in exosome biogenesis, along with the species-specific protein tyrosine phosphatase PRL-1 (PRL-1). This image was generated using multiple software as described in materials and methods.

Our findings provide valuable insight and advancements in the identification of EVs of non-mammalian origin, challenging the use of certain commonly used markers in the identification of *Leishmania* spp.*-*derived extracellular vesicles/exosomes. Moreover, we describe potential new exosome production-related markers (ESCRT-related), like VPS37, and parasite-specific markers like PRL-1, which may enhance the specificity of EV identification or isolation procedures, particularly for *Leishmania* spp.*-*derived exosomes (summarized in **Figure 6**). Follow-up studies to this exploratory work may provide valuable insight into the sensitivity of these markers in immunohistochemistry.

## Data availability statement

The datasets presented in this study can be found in online repositories. The names of the repository/repositories and accession number(s) can be found below: The data has now been deposited in EBI PRIDE. Project accession: PXD048015 Project DOI: 10.6019/PXD048015.

## Author contributions

AS: Conceptualization, Data curation, Formal Analysis, Methodology, Validation, Writing – original draft, Writing – review & editing, Investigation, Visualization. AL: Writing – review & editing, Data curation, Formal Analysis, Investigation, Visualization. MM-P: Software, Visualization, Writing – review & editing, Conceptualization, Data curation, Formal Analysis, Investigation, Methodology. MO: Conceptualization, Funding acquisition, Investigation, Methodology, Project administration, Resources, Supervision, Validation, Writing – review & editing, Data curation, Visualization.
