# Supplementary material for: Identification of potential novel proteomic markers of Leishmania spp.-derived exosomes
Source: Front Cell Infect Microbiol. 2024 Feb 19;14:1354636. doi: 10.3389/fcimb.2024.1354636 (PMC10910114; doi:10.3389/fcimb.2024.1354636)
Supplement: Supplementary file 1 [file Presentation_1.pdf]

Supplementary Material

Supplementary Figures

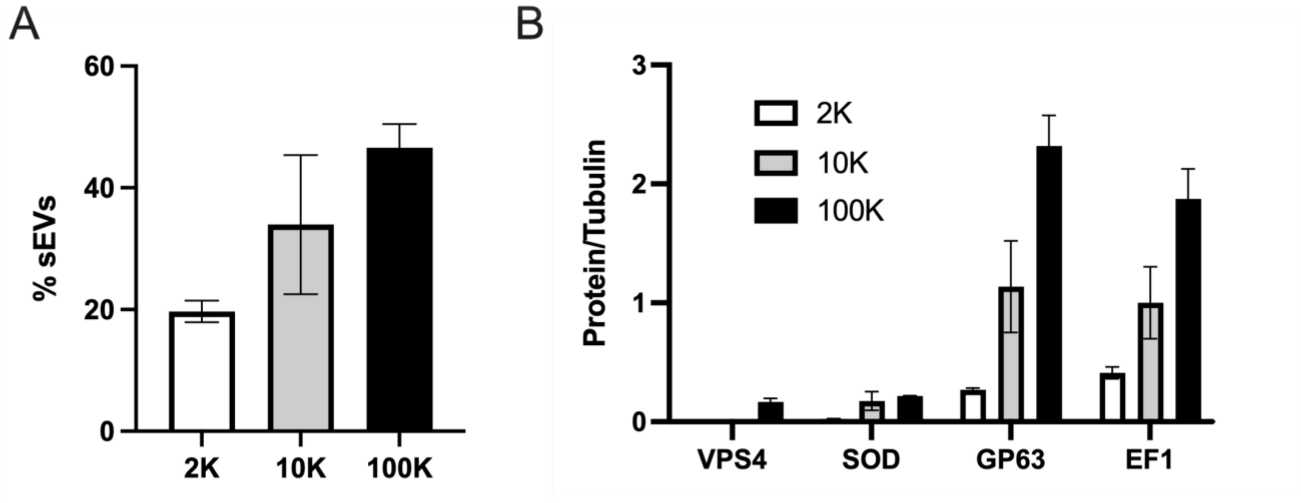

Supplementary Figure 1. Relative content of probable exosomes and of exosomal proteins identified in fractions of *Leishmania major*-derived extracellular vesicles. (A) Size distribution and concentration of extracellular vesicle populations were determined with NTA using triplicates for each fraction (*n=3*). Small EVs (sEVs) are defined as EVs with a diameter inferior to 150 nm (instrumental error ± 50 nm). **(B)** The protein content of *Leishmania major*-derived EVs was catalogued by mass spectrometry (*L. major* database) using triplicates for each fraction (*n=3*) and analyzed using Mascot followed by Scaffold Software. Peptide counts for canonical EV proteins were normalized to peptide counts for tubulin.

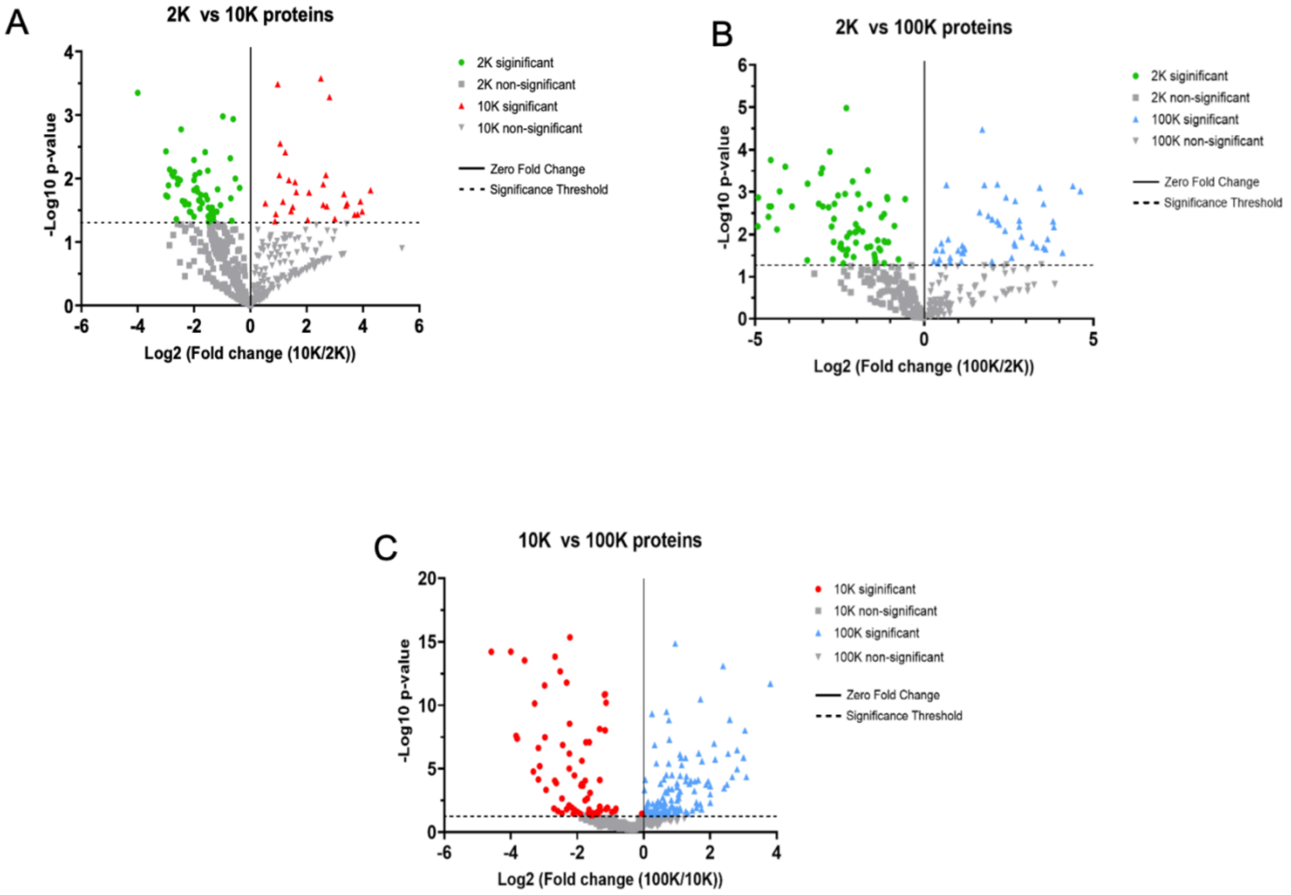

Supplementary Figure 2. Comparative volcano plot of proteins identified in fractions of *Leishmania major*-derived extracellular vesicles. The protein content of *Leishmania major*-derived EVs was catalogued by mass spectrometry (*L. major* database) using triplicates for each fraction ($n=3$) and analyzed using Mascot followed by Scaffold Software. Quantitative analysis was performed using 2-way ANOVA and plotted according to fold change and statistical significance. Volcano plots represent the expression profiles of proteins identified in the **(A)** 2K vs. 10K fractions, **(B)** 2K vs. 100K fractions, and **(C)** 10K vs. 100K fractions. Coloured dots indicate statistically significant differences in expression ($p<0.05$) between the groups.

Supplementary Tables

	2K			10K			100K		
	N˚ Genes	% Genes	% Function	N˚ Genes	% Genes	% Function	N˚ Genes	% Genes	% Function
**Response to stimulus (GO:0050896)**	12	3.90%	3.90%	19	4.80%	4.60%	16	5.40%	4.90%
**Signaling (GO:0023052)**	5	1.60%	1.60%	10	2.50%	2.40%	9	3.00%	2.80%
**Cellular process (GO:0009987)**	144	47.20%	47.40%	192	48.40%	46.00%	146	49.20%	45.10%
**Metabolic process (GO:0008152)**	87	28.50%	28.60%	121	30.50%	29.00%	87	29.30%	26.90%
**Locomotion (GO:0040011)**	3	1.00%	1.00%	1	0.30%	0.20%	0	0.00%	0.00%
**Biological regulation (GO:0065007)**	24	7.90%	7.90%	34	8.60%	8.20%	25	8.40%	7.70%
**Localization (GO:0051179)**	29	9.50%	9.50%	40	10.10%	9.60%	41	13.80%	12.70%

Supplementary Table 1. Classification of total proteins detected in fractions of *Leishmania major*-derived extracellular vesicles according to Biological Process Gene Ontology Annotation. Gene Ontology analysis was performed on LC-MS/MS proteomic datasets (*L. major* database) of all identified proteins in 2K, 10K and 100K fractions of EVs derived from *L. major*. PANTHER database (pantherdb.org) was used to annotate protein sets according to biological processes, providing the number of genes associated to diverse functions (N˚ Genes), the percentage of total genes associated with each function (% Genes), and the percentage of the total function that is covered by the protein list (% Function)

	2K			10K			100K		
	N˚ Genes	% Genes	% Function	N˚ Genes	% Genes	% Function	N˚ Genes	% Genes	% Function
**Translation regulator activity (GO:0045182)**	5	1.60%	2.30%	8	2.00%	2.90%	4	1.30%	1.90%
**Binding (GO:0005488)**	78	25.60%	36.10%	92	23.20%	33.00%	62	20.90%	29.00%
**Structural molecule activity (GO:0005198)**	13	4.30%	6.00%	12	3.00%	4.30%	8	2.70%	3.70%
**Molecular function regulator (GO:0098772)**	1	0.30%	0.50%	4	1.00%	1.40%	4	1.30%	1.90%
**Catalytic activity (GO:0003824)**	110	36.10%	50.90%	151	38.00%	54.10%	121	40.70%	56.50%
**Transporter activity (GO:0005215)**	9	3.00%	4.20%	12	3.00%	4.30%	15	5.10%	7.00%

Supplementary Table 2. Classification of total proteins detected in fractions of *Leishmania major*-derived extracellular vesicles according to Molecular Function Gene Ontology Annotation. Gene Ontology analysis was performed on LC-MS/MS proteomic datasets (*L. major* database) of all identified proteins in 2K, 10K and 100K fractions of EVs derived from *L. major*. PANTHER database (pantherdb.org) was used to annotate protein sets according to molecular functions, providing the number of genes associated to diverse functions (N˚ Genes), the percentage of total genes associated with each function (% Genes), and the percentage of the total function that is covered by the protein list (% Function)

	2K			10K			100K		
	N˚ Genes	% Genes	% Function	N˚ Genes	% Genes	% Function	N˚ Genes	% Genes	% Function
**Mitochondrion (GO:0005739)**	16	29.60%	26.70%	10	15.90%	14.10%	4	7.00%	6.30%
**Vacuole (GO:0005773)**	16	29.60%	26.70%	19	30.20%	26.80%	23	40.40%	35.90%
**Microbody (GO:0042579)**	1	1.90%	1.70%	1	1.60%	1.40%	1	1.80%	1.60%
**Endoplasmic reticulum (GO:0005783)**	4	7.40%	6.70%	7	11.10%	9.90%	6	10.50%	9.40%
**Golgi apparatus (GO:0005794)**	3	5.60%	5.00%	4	6.30%	5.60%	3	5.30%	4.70%
**Endoplasmic reticulum-Golgi intermediate compartment (GO:0005793)**	1	1.90%	1.70%	1	1.60%	1.40%	1	1.80%	1.60%
**Nucleus (GO:0005634)**	19	35.20%	31.70%	29	46.00%	40.80%	26	45.60%	40.60%

Supplementary Table 3. Classification of total proteins detected in fractions of *Leishmania major*-derived extracellular vesicles according to Cellular Component Gene Ontology Annotation. Gene Ontology analysis was performed on LC-MS/MS proteomic datasets (*L. major* database) of all identified proteins in 2K, 10K and 100K fractions of EVs derived from *L. major*. PANTHER database (pantherdb.org) was used to annotate protein sets according to cellular components (Level 3 – Membrane bound organelle), providing the number of genes associated to diverse cellular components (N˚ Genes), the percentage of total genes associated with each cellular component (% Genes), and the percentage of the total cellular component that is covered by the protein list (% Function)

Protein Name	Accession N° (UniProt)	Molecular Weight	2K Average Peptide Count	10K Average Peptide Count	100K Average Peptide Count
GP63, leishmanolysin	Q4QHH1_LEIMA	64 kDa	68.7 ± 1.2	127.6 ± 15.4	151.3 ± 19.4
Elongation factor 1-alpha	Q4QEI9_LEIMA	49 kDa	104.7 ± 6.6	116.3 ± 16.2	119.3 ± 5.2
Enolase	Q4QFL8_LEIMA	46 kDa	14.3 ± 2.7	44.3 ± 5.5	57.6 ± 5.5
Heat shock protein 83-1	E9AHM8_LEIIN	79 kDa	57.7 ± 3.7	78.0 ± 16.4	63.6 ± 7.4
Tryparedoxin	E9ADX4_LEIMA	17 kDa	1.3 ± 1.1	4.6 ± 2.0	7.0 ± 3.3
Heat-shock protein hsp70	Q4Q7Y4_LEIMA	72 kDa	84.0 ± 4.9	141.0 ± 18.0	114.0 ± 12.4
Surface antigen protein	Q4QGL8_LEIMA	65 kDa	11.0 ± 0.5	11.6 ± 1.9	17.3 ± 0.3
Calpain-like cysteine peptidase	Q4QCS8_LEIMA	91 kDa	4.0 ± 0.5	20.0 ± 8.2	9.0 ± 3.7
14-3-3 protein	A4HUU6_LEIIN	29 kDa	4.3 ± 0.3	7.3 ± 3.1	9.0 ± 2.6

Supplementary Table 4. Expression levels of proteins commonly used as EV markers of *Leishmania spp*. The protein content of *Leishmania major*-derived EVs was catalogued by mass spectrometry (*L. major* database) using triplicates for each fraction (*n=3*) and analyzed using Mascot followed by Scaffold Software. Numbers indicate the average peptide count ± the standard error to the mean (SEM). HSP83 and HSP70 were detected in all three EV fractions at similar levels of expression. GP63, Enolase, and Surface Antigen Protein levels increased in expression in smaller EVs, reaching

their highest level in the 100K fraction. EF-1, Tryparedoxin peroxidase, Calpain-like cysteine peptidase, and 14-3-3 protein did not significantly vary between the three EV groups.

Protein Name	Accession # (UniProt)	Molecular Weight
2-methoxy-6-polyprenyl-1,4-benzoquinol methylase, mitochondrial	Q4Q0M6_LEIMA	32 kDa
Putative 60S ribosomal protein L10a	Q4QDX9_LEIMA	25 kDa
Uncharacterized protein	Q95Z90_LEIMA	67 kDa
Uncharacterized protein	Q4Q867_LEIMA	81 kDa
Uncharacterized protein	Q4Q6S0_LEIMA	51 kDa
Uncharacterized protein	Q4QI57_LEIMA	97 kDa
Uncharacterized protein	E9ADV8_LEIMA	67 kDa
Uncharacterized protein	Q4Q3U0_LEIMA	43 kDa
Uncharacterized protein	Q4Q6F8_LEIMA	39 kDa
Putative fumarate hydratase	E9AWM1_LEIMU	61 kDa
Uncharacterized protein	A4I3M7_LEIIN	14 kDa
Uncharacterized protein	Q4Q9Q7_LEIMA	90 kDa
Uncharacterized protein	Q4Q7B3_LEIMA	590 kDa
Uncharacterized protein	Q4Q754_LEIMA	410 kDa
Cation-transporting ATPase	Q4QII2_LEIMA	139 kDa
Uncharacterized protein	Q4Q801_LEIMA	193 kDa
Uncharacterized protein	Q4Q2U2_LEIMA	477 kDa
Polyadenylate-binding protein	A0A088S1M1_9TRYP	62 kDa
Aromatic amino acid hydroxylase-like	Q6WRI4_LEIMA	51 kDa
Uncharacterized protein	Q4QCT5_LEIMA	124 kDa
Uncharacterized protein	Q4Q0Y7_LEIMA	170 kDa
Uncharacterized protein	Q4QCC9_LEIMA	347 kDa
Putative poly-zinc finger protein 2	Q4Q1R1_LEIMA	15 kDa
Uncharacterized protein	Q4Q353_LEIMA	38 kDa
Putative chaperonin HSP60/CNP60	Q4Q711_LEIMA	58 kDa
Heat shock protein HslVU, ATPase subunit HslU, putative	A0A088RNE1_9TRYP	48 kDa
Uncharacterized protein	Q4Q0C0_LEIMA	84 kDa
Uncharacterized protein	Q4Q0Q2_LEIMA	105 kDa
Putative vacuolar ATP synthase catalytic subunit An	A0A0N1I8L6_LEPSE	68 kDa
Putative histone H3 variant	Q4QDF8_LEIMA	16 kDa
Uncharacterized protein	Q4QJ60_LEIMA	62 kDa
Uncharacterized protein	Q4Q5Q8_LEIMA	140 kDa
Putative mitochondrial RNA binding protein	Q4QB87_LEIMA	40 kDa

Putative heat shock protein-like protein	Q4Q584_LEIMA	36 kDa
Uncharacterized protein	Q4Q3W0_LEIMA	60 kDa
Uncharacterized protein	Q4Q483_LEIMA	28 kDa
Hypothetical predicted multi-pass transmembrane protein	Q4QAE6_LEIMA	44 kDa
Putative pumilio protein 6	Q4Q475_LEIMA	93 kDa
Uncharacterized protein	Q4Q0N7_LEIMA	388 kDa
Uncharacterized protein	Q4Q0F7_LEIMA	261 kDa
Uncharacterized protein	Q4QGM3_LEIMA	91 kDa
Histone H3	H3_LEIIN	15 kDa
Dynein intermediate-chain-like protein	Q4QAV3_LEIMA	75 kDa
3-hydroxy-3-methylglutaryl coenzyme A reductase	A4I602_LEIIN	46 kDa
Uncharacterized protein	Q4Q3D8_LEIMA	358 kDa
Uncharacterized protein	Q4QHG5_LEIMA	707 kDa
Uncharacterized protein	Q4QIW6_LEIMA	33 kDa
Putative serine/threonine-protein kinase	Q4Q6T0_LEIMA	85 kDa
Uncharacterized protein	Q4QBA6_LEIMA	116 kDa
3-methylcrotonoyl-CoA carboxylase beta subunit, putative	E9BAP8_LEIDB	58 kDa
Uncharacterized protein	Q4Q9Z4_LEIMA	84 kDa
Uncharacterized protein	Q4Q0J5_LEIMA	78 kDa
Uncharacterized protein	Q4QG62_LEIMA	162 kDa
Uncharacterized protein	Q4QH73_LEIMA	148 kDa
Putative 2-oxoisovalerate dehydrogenase beta subunit, mitochondrial	E9AEI2_LEIMA	40 kDa
Uncharacterized protein	Q4QEJ9_LEIMA	493 kDa
Putative electron-transfer-flavoprotein, alpha polypeptide	Q4Q8F2_LEIMA	34 kDa
Uncharacterized protein	E9ADA0_LEIMA	46 kDa
ADP/ATP mitochondrial carrier-like protein	Q4QFN6_LEIMA	41 kDa
Histone H2B	E9AR94_LEIMU	12 kDa
Uncharacterized protein	Q4QBQ7_LEIMA	101 kDa
Putative ATP-binding cassette protein subfamily C, member 2	Q4QBF3_LEIMA	174 kDa
LETM1 and EF-hand domain-containing protein 1, putative	A0A088SE62_9TRYP	57 kDa
Poly(A)-binding protein 3	Q4QA88_LEIMA	61 kDa
Uncharacterized protein	Q4Q3G7_LEIMA	47 kDa
Putative kinesin K39	Q4QFM2_LEIMA	341 kDa
Uncharacterized protein	Q4Q360_LEIMA	30 kDa
Beta-tubulin	A4HLD6_LEIBR	21 kDa

Uncharacterized protein	A4I7H7_LEIIN	112 kDa
Uncharacterized protein	Q4QIP4_LEIMA	539 kDa

Supplementary Table 5. Unique proteins identified in the 2K fraction of *Leishmania major* extracellular vesicles. The protein content of *Leishmania major*-derived EVs was catalogued by mass spectrometry (*L. major* database) using triplicates for each fraction ($n=3$) and analyzed using Mascot followed by Scaffold Software. Unique proteins detected by LC-MS/MS in each fraction were determined using inclusion criteria of a minimum of 2 detected peptides, a peptide threshold of 80%, and a protein identity of 95%, and excluded if they represented a true hit in more than one group. 70 proteins were identified as unique to the 2K fraction.

Protein Name	Accession # (UniProt)	Molecular Weight
Uncharacterized protein	Q4QBP4_LEIMA	129 kDa
Putative glucosamine-fructose-6-phosphate aminotransferase	Q4QIY2_LEIMA	73 kDa
Uncharacterized protein	Q4QEJ8_LEIMA	88 kDa
Acetylornithine deacetylase-like protein	Q4QIR7_LEIMA	43 kDa
Uncharacterized protein	A4HK11_LEIBR	181 kDa
Uncharacterized protein	Q4Q1S2_LEIMA	98 kDa
Arginase	A0A145YEM0_LEIMA	36 kDa
Putative 40S ribosomal protein S10	Q4Q1X7_LEIMA	19 kDa
Eukaryotic translation initiation factor 3 subunit I	Q4Q127_LEIMA	45 kDa
Putative cytochrome-b5 reductase	A4I862_LEIIN	37 kDa
60s ribosomal protein L30	A0A0N1PD37_LEPSE	11 kDa
Putative cAMP-specific phosphodiesterase	Q95Z92_LEIMA	79 kDa
Ubiquitin-conjugating enzyme-like protein	E9BBK6_LEIDB	16 kDa
Putative phosphomevalonate kinase protein	Q4QF34_LEIMA	49 kDa
Putative glutaminyl-tRNA synthetase	Q4QF36_LEIMA	66 kDa
Cytochrome c oxidase subunit I	A4ICE1_LEIIN	14 kDa
Translation initiation factor-like protein	A0A088S320_9TRYP	38 kDa
Uncharacterized protein	Q4Q279_LEIMA	89 kDa
Uncharacterized protein	Q4QA76_LEIMA	151 kDa
Uncharacterized protein	Q4Q3P1_LEIMA	69 kDa
Uncharacterized protein	E9B3A4_LEIMU	24 kDa
Uncharacterized protein	Q4Q1J4_LEIMA	50 kDa
Phosphodiesterase	Q6S998_LEIMA	71 kDa
Putative ubiquitin carboxy-terminal hydrolase	Q9U1E5_LEIMA	148 kDa
Putative intraflagellar transport protein IFT88	E9AD91_LEIMA	91 kDa
Uncharacterized protein	Q4QEE6_LEIMA	60 kDa
Uncharacterized protein	Q4QF65_LEIMA	138 kDa
Uncharacterized protein	A4HUJ0_LEIIN	46 kDa
Uncharacterized protein	Q4Q201_LEIMA	138 kDa
Putative ubiquitin-protein ligase	Q4Q0C6_LEIMA	455 kDa
Putative proteasome regulatory non-ATP-ase subunit 2	Q4Q893_LEIMA	106 kDa
Acidocalcisomal pyrophosphatase	Q4QH59_LEIMA	51 kDa
Uncharacterized protein	Q4QE99_LEIMA	55 kDa
Putative calpain-like cysteine peptidase	A4HS39_LEIIN	95 kDa

Uncharacterized protein	E9AFI1_LEIMA	133 kDa
Putative casein kinase II	A4IB73_LEIIN	43 kDa

Supplementary Table 6. Unique proteins identified in the 10K fraction of *Leishmania major* extracellular vesicles. The protein content of *Leishmania major*-derived EVs was catalogued by mass spectrometry (*L. major* database) using triplicates for each fraction (*n=3*) and analyzed using Mascot followed by Scaffold Software. Unique proteins detected by LC-MS/MS in each fraction were determined using inclusion criteria of a minimum of 2 detected peptides, a peptide threshold of 80%, and a protein identity of 95%, and excluded if they represented a true hit in more than one group. 36 proteins were identified as unique to the 10K fraction.

Protein Name	Accession # (UniProt)	Molecular Weight
Putative calcium motive p-type ATPase	E9AF31_LEIMA	122 kDa
Uncharacterized protein / *IST1*	Q4Q9F3_LEIMA	30 kDa
Qc-SNARE protein	Q4QIG6_LEIMA	24 kDa
SNF7	E9AFR7_LEIMA	24 kDa
Succinyl-diaminopimelate desuccinylase-like protein	A4I6X9_LEIIN	51 kDa
Uncharacterized protein	Q4QH03_LEIMA	22 kDa
Protein tyrosine phosphatase-like protein / *PRL1*	Q4QEZ7_LEIMA	19 kDa
Nucleobase transporter	Q4QG33_LEIMA	55 kDa
Putative ATP-binding cassette protein subfamily A, member 8	E9AD74_LEIMA	202 kDa
SNF-7-like protein	E9AEN3_LEIMA	26 kDa
VTA1	Q4QJC8_LEIMA	39 kDa
Uncharacterized protein / *CHMP2B*	Q4QB64_LEIMA	81 kDa
Uncharacterized protein / *DNA Pol III*	Q4QH65_LEIMA	94 kDa
Uncharacterized protein	Q4QB65_LEIMA	38 kDa
Sucrose-phosphate synthase-like protein	Q4QES5_LEIMA	52 kDa
Uncharacterized protein / *ALIX*	E9ADE3_LEIMA	102 kDa
Uncharacterized protein	Q4Q276_LEIMA	17 kDa
Uncharacterized protein	Q9N852_LEIMA	54 kDa
VPS37	Q4Q8L4_LEIMA	32 kDa
Putative serine/threonine protein phosphatase type 5	Q4QE27_LEIMA	53 kDa
Uncharacterized protein	Q4Q6I3_LEIMA	65 kDa
Putative 2,4-dihydroxyhept-2-ene-1,7-dioic acid aldolase	Q4Q9N8_LEIMA	30 kDa
Phosphodiesterase	E9AEA6_LEIMA	81 kDa
Putative valyl-tRNA synthetase	Q4Q6X7_LEIMA	110 kDa
Uncharacterized protein / *LRR-RI*	Q4QAY9_LEIMA	33 kDa
Putative phosphatase 2C	A4I565_LEIIN	42 kDa
Glucose transporter, lmgt3	Q4Q0D2_LEIMA	62 kDa
Putative amino acid transporter aATP11	Q4Q6M9_LEIMA	52 kDa
Putative folate/biopterin transporter	Q4QHI0_LEIMA	75 kDa
Formate--tetrahydrofolate ligase	A4I5T5_LEIIN	67 kDa
Uncharacterized protein / *AMMECR1*	Q4Q5M0_LEIMA	21 kDa
Putative folate/biopterin transporter	Q4QHI2_LEIMA	73 kDa

Supplementary Table 7. Unique proteins identified in the 100K fraction of *Leishmania major* extracellular vesicles. The protein content of *Leishmania major*-derived EVs was catalogued by mass spectrometry (*L. major* database) using triplicates for each fraction (*n=3*) and analyzed using Mascot followed by Scaffold Software. Unique proteins detected by LC-MS/MS in each fraction were determined using inclusion criteria of a minimum of 2 detected peptides, a peptide threshold of 80%, and a protein identity of 95%, and excluded if they represented a true hit in more than one group. 32 proteins were identified as unique to the 100K fraction. Homologous protein names were manually searched using UniProt for uncharacterized proteins and have been indicated in italics when possible.
